# Selection for Male Weapons Boosts Female Fecundity, Eliminating Sexual Conflict in the Bulb Mite

**DOI:** 10.1038/s41598-020-59254-2

**Published:** 2020-02-11

**Authors:** Bruno A. Buzatto, Huon L. Clark

**Affiliations:** 1 Centre for Evolutionary Biology, School of Biological Sciences (M092), The University of Western Australia, 35 Stirling Highway, Crawley, 6009 WA Australia; 2 Department of Biological Sciences (E8C 209), Macquarie University, Eastern Road, Sydney, 2109 NSW Australia

**Keywords:** Evolutionary genetics, Sexual selection

## Abstract

Extreme differences between the sexes are usually explained by intense sexual selection on male weapons or ornaments. Sexually antagonistic genes, with a positive effect on male traits but a negative effect on female fitness, create a negative inter-sexual correlation for fitness (sexual conflict). However, such antagonism might not be apparent if sexually selected male traits are condition-dependent, and condition elevates female fitness. Here we reveal a surprising positive genetic correlation between male weaponry and female fecundity. Using mite lines that had previously been through 13 generations of selection on male weapons (fighting legs), we investigated correlated evolution in female fecundity. Females from lines under positive selection for weapons (up lines) evolved higher fecundity, despite evolving costly, thicker legs. This is likely because male mites have condition-dependent weaponry that increases our ability to indirectly select on male condition. Alleles with positive effects on condition in both sexes could have generated this correlation because: the up lines evolved a higher proportion of fighters and there were positive correlations between weapon size and the male morph and sex ratios of the offspring. This positive inter-sexual genetic correlation should boost the evolution of male weapons and extreme sex differences.

## Introduction

Sexual dimorphism intrigued Darwin, and led to the establishment of the field of sexual selection that continues to fascinate scientists. Morphological dimorphisms are relatively well understood when manifest between the sexes^1,2^, but discrete morphs also exist within a sex, usually between alternative male phenotypes that employ different tactics to gain reproductive success^3^. Although male polymorphisms are widespread in many animal groups^3^, their evolution is far less well understood. Expression of these alternative phenotypes is frequently conditionally dependent on environmental factors^4^. This conditionality, allied to the fact that intrasexual dimorphism cannot be regulated by sex chromosomes^2,5,6^, makes such dimorphism intriguing from an evolutionary perspective.

Although alternative male phenotypes are often radically different, they can be expressed by individuals with almost identical genomes, making them an extreme case of phenotypic plasticity, known as polyphenism^7^. The literature is rife with claims about ‘all-or-none’ physiological mechanisms that decouple the developmental pathway of different male phenotypes so that they each evolved independently^8^. Although such mechanisms have been elucidated for polyphenic dung beetles^9^, the putative ability of male phenotypes to evolve independently has been criticised as an oversimplification, and empirical studies have exposed strong genetic correlations between different male phenotypes^10^. This sets the scene for a situation whereby genes that improve the fitness of, say, a fighter morph might lower that of a sneaker morph. This type of evolutionary conflict has been termed intra-locus tactical conflict, in reference to the different mating tactics adopted by different male phenotypes^11^.

**Figure 1:**
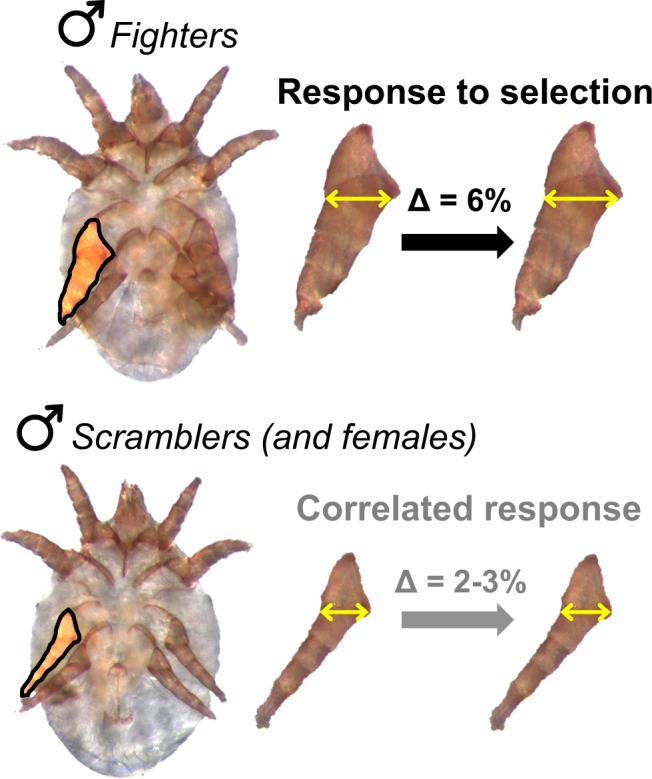
Morph-specific, bidirectional artificial selection on weapon size (relative width of the third pair of fighting legs, highlighted) of *Rhizoglyphus echinopus* fighters caused a divergence of about 6% (yellow arrows) after nine generations. Selection was only applied on fighters, but scramblers (the other male morph) and females (not shown) showed a correlated response of about 2–3%, even though they lack fighting modifications in their third pair of legs. Images are manipulated to show the magnitude of difference; original data from Buzatto *et al*.^12^.

We recently performed the first direct test of whether selection targeting one male morph affects the evolution of another. Fighter males of the bulb mite *Rhizoglyphus echinopus* possess a modified third pair of legs that are twice as thick as the other legs, and sharply terminated. These legs are used in fatal fights. Smaller nymphs never develop such leg modifications as adults, and instead become *scramblers* that wander around the colonies searching for unguarded females. We previously applied artificial selection to the modified legs of fighters, and as a consequence we observed the coevolution of relative leg width in both scrambler males and females (Fig. 1) in less than ten generations (results reported in^12^). That effect, driven by genetically correlated responses, was remarkably similar in magnitude in scramblers and females^12^.

**Figure 2:**
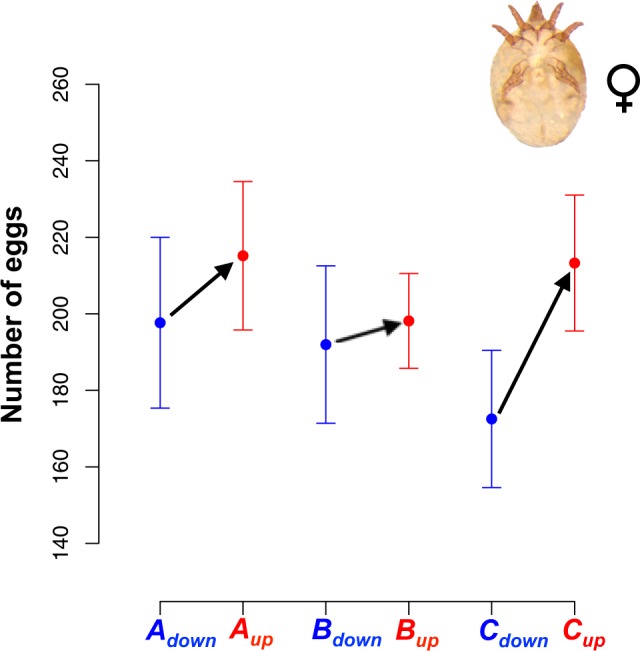
Correlated evolution of female fecundity in response to artificial selection on male weapons of the bulb mite *Rhizoglyphus echinopus*: lines selected for larger weapons (up lines, in red) evolved higher fecundity than lines selected for smaller weapons (down lines, in blue). This correlated response was significant (ΔAIC_c_ = 2.81 between two models that only differed in having selection direction as a fixed effect; see Methods) and occurred despite females lacking fighting legs, and selection only being applied to male fighters.

This correlated evolution has the potential to lower the efficacy of scrambler males, because thicker legs are costly to produce^13^, and hinder locomotion^14^. If these costs are also present in females, the expression of genes that affect relative third leg width could decrease female lifetime fecundity, generating sexual conflict (as in^15,16^). Instead, we now found that lines selected for thicker legs in fighter males (up lines) evolved higher female fecundity than down lines selected for thinner legs in fighter males. Females from the up lines evolved higher fecundity (Fig. 2), albeit to variable degrees, across all three replicates. We hypothesise that the evolution of greater fecundity is due to a genetically correlated response: genes that increase relative leg width in fighter males somehow improve female fecundity as a side effect, perhaps via epistasis. It is of course also possible that selection on relative leg width in males resulted in correlated evolution of aggression, or any other male or female behaviour that might underlie the effects we observed on female fecundity. It would be very interesting to investigate this hypothesis in the future.

Our finding sharply contrasts with work on another arthropod, the broad-horned flour beetle, where artificial selection for a larger male-only trait (mandible horns) lowered female fecundity, revealing clear sexual conflict^15^. Unlike the bulb mites, however, in these beetles males are not polyphenic. In a congeneric, and also male dimorphic mite, *Rhizoglyphus robini*, artificial selection for fighters (by only allowing fighters to mate) reduced the fecundity and longevity of females, again revealing intra-locus sexual conflict with genes for traits favoured in the male fighter morph^16^. Our protocol was different in a crucial aspect, however, because we selected on weapon size in the fighter morph, rather than simply selecting for one of the morphs. Our main result suggests not only that sexual conflict related to male weaponry is eliminated, but also that selection on male weapon enhances female fitness. This could be driven by a positive genetic correlation between a costly secondary sexual trait in males^13^ and female fitness, which would accelerate the evolution of extreme growth in sexually selected traits, rather than constrain it. But how does weapon size and female fecundity become genetically correlated?

We hypothesize that the co-evolution of male weapon size and female fecundity resulted from selection of alleles that elevate condition. Condition is defined as the ability to acquire, assimilate and allocate resources to life history traits. It is partly determined by the many genes that affect traits for resource acquisition and utilization (e.g. foraging, immunity, digestion), resulting in a large mutation target that could readily maintain genetic variation in traits under strong directional sexual selection^17^. Many secondary sexually selected traits, including weapons, show far stronger condition-dependent expression^18^ than other male traits^19^. And fecundity is usually higher for females in good condition^20^. In that case, it is possible that our selection protocol, which targeted a costly, condition-dependent weapon, favoured alleles that affect condition. We suggest that this could have caused our up lines to evolve better condition than our down lines, thereby elevating condition-dependent female fecundity.

**Figure 3:**
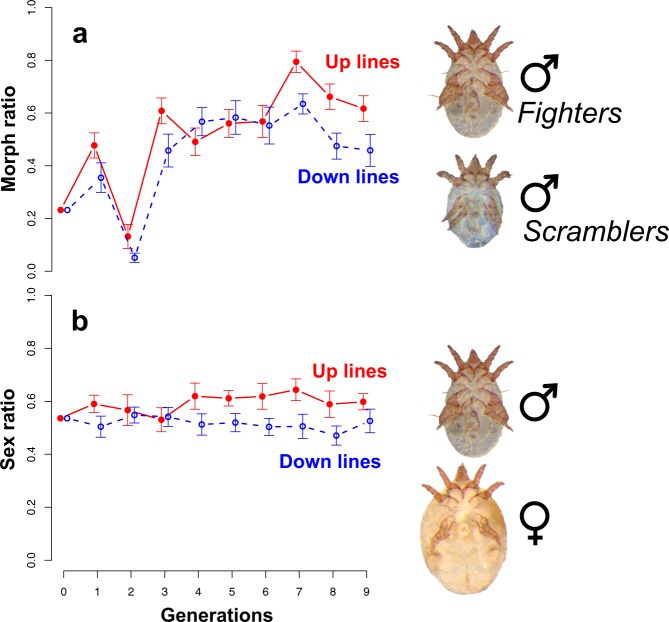
The evolution of (**a**) the proportion of male fighters and (**b**) the sex ratio (proportion of males) in the bulb mite *Rhizoglyphus echinopus*, for nine generations of morph-specific selection for larger (up lines; red full circles and solid lines) and smaller weapons (down lines; blue open circles and dashed lines). Means and standard errors are from approximately 5 families per generation per line (total: 272 families, 4,257 males and 3,408 females).

Support for the hypothesis that condition-dependence drives our results comes from the observed evolution of sex and morph ratios (proportion of fighter males) in our artificially selected lines (Fig. 3). We expected an increase in the proportion of fighter males in all our lines, since fighter males were always used as sires, but we did not expect a significant effect of selection direction on morph ratio. This is because current understanding of male dimorphism in *R. echinopus* suggests that the threshold body size that an individual has to attain to express the fighter phenotype (an individual’s genetic threshold) is genetically independent to those that affect male body size itself^21^. Consequently, artificial selection on fighter relative leg width should not have affected morph ratio in our selection lines. We also had no reason to expect our selection protocols to alter the sex ratio. Nonetheless, both the morph and offspring sex ratio evolved in a direction that supports our hypothesis that selection on weapons targets genes for good condition.

As expected, the proportion of fighters steadily increased across the generations from 0.23 to 0.62 in the up lines and to 0.46 in the down lines (Fig. 3a). There was no single best model and the two top models performed equally well (models 1 and 2 in Table 1). They contained the fixed effects of *generation*, and the *direction of selection*, probably due to the fact that morph ratio was slightly higher in the up lines during most of the experiment (except generations 4 and 5; Fig. 3a). Despite the conditionality of male dimorphism in *R. echinopus*, there is genetic variation for the threshold body size that has to be attained to trigger the fighter phenotype^22^, conferring some heritability to morph expression. Acarid mites have varying degrees of morph heritability in different species^23^, but selection on morph type seems to always cause morph ratio evolution^16,24^. The small but significant difference in the morph ratio between the up and down lines (Fig. 3a) in our study was, however, surprising. Our *a posteriori* explanation for it fits our hypothesis to explain the coevolution of female fecundity — by selecting for greater relative third leg width in the up lines, we selected on genes for condition since these legs are costly and show condition-dependent expression^14^.

**Table 1:** The effects of generation and selection direction on morph and sex ratio in a mite.

Rank	Fixed effects	*k*	AIC_c_	ΔAIC_c_	Weight	Log-likelihood	Cumulative weight
***Morph ratio***							
1	Generation + Direction	4	1404.8	—	0.67	−698.3	0.67
2	Generation + Direction + (Generation x Direction)	5	1406.3	1.5	0.31	−698.0	0.98
3	Generation	3	1412.2	7.4	0.02	−703.1	1.00
4	Direction	3	1452.6	47.8	0.00	−723.3	1.00
5	*Null*	2	1458.5	53.7	0.00	−727.2	1.00
***Sex ratio***							
1	Direction	3	1579.9	—	0.61	−786.9	0.61
2	Generation + Direction	4	1581.9	2	0.23	−786.9	0.83
3	Generation + Direction + (Generation x Direction)	5	1582.5	2.6	0.17	−786.1	1.00
4	*Null*	2	1596.5	16.6	0.00	−796.3	1.00
5	Generation	3	1598.5	18.6	0.00	−796.2	1.00

The analyses used model selection considering the effects of generation, selection direction (up or down), and their interaction on morph and sex ration in the bulb mite Rhizoglyphus echinopus. All models are binomial GLMMs with an observation-level random effect to deal with overdispersion.

The proportion of sons per family was higher in the up lines in almost all generations (Fig. 3b), increasing from 0.54 to 0.60 throughout the experiment, whereas in the down lines this proportion remained relatively unchanged, averaging 0.52 across the generations. The top ranked model only contained the effect of *selection direction* (Table 1). The larger proportion of males in the up lines (Fig. 3b) was also surprising, given that *R. echinopus* seems to possess chromosomal (XX/XY) sex determination^25^. If our selection protocol really selected on male condition, the male-biased sex ratio in up lines could reflect strategic sex allocation by females. According to Trivers-Willard hypothesis, since males produced by females in better condition have greater reproductive success than males produced by females in poorer conditions, females in better condition should invest relatively more in producing male offspring^26^.

If relative leg width in *R. echinopus* fighters indicates better condition, it is also possible that females produce more sons when mated to males with thicker legs. There is generally weak support for strategic maternal sex allocation in response to sire quality^27^, but some evidence for this exists in other groups of mites^28^. *Post-hoc* support for this idea comes from the positive correlations between sire relative leg width and offspring morph (Spearman, r_*s*_ = 0.208, *p-value* < 0.001, n = 256 families) and sex (Spearman, r_*s*_ = 0.288, *p-value* < 0.0001, n = 256 families) ratios. Importantly, this only holds true if male offspring, and more specifically fighter males, return higher fitness for the parents than female or scrambler male offspring. Moreover, we did not expect these correlations *a priori*, so we do not present them here as formal hypotheses tests, but merely descriptions of interesting patterns that inspired our speculations.

In conclusion, our key finding was that bulb mites do not experience sexual conflict during the evolution of a male-dimorphic weapon because of a genetic correlation between larger male secondary sexual traits and greater female fecundity. This appears to arise because both traits are positively affected by condition, and selection for condition-dependent weapon size indirectly favours genes that increase condition. This positive inter-sex genetic correlation should facilitate the evolution of weaponry and extreme sexual dimorphism. Alternatively, it is possible that intralocus sexual conflict for the fighting legs is completely resolved by sexual dimorphism in this trait, but not for other traits associated with the alternative male tactics. If females are actually more in conflict with a trait of the scrambler morph, and if this trait was favored by our selection for thinner legs (typical of scramblers) in the down lines, this could also explain the lowered fecundity in females from these lines. One way or the other, our results indicate that the costliness of male secondary sexual traits does not inevitably lead to intra-locus sexual conflict, even if selection on this trait causes correlated evolution in the homologous trait of females. These implications probably apply to ornaments as well, despite the difference between the signalling function of ornaments and the fighting function of weapons^29^. Direct selection on weapon size might simultaneously favour genes for good condition, even if that weapon is not used in any context as a signal of good condition, as long as the weapon is condition dependent in its expression^18^.

## Methods

From May 2016 to October 2017, we imposed bidirectional, morph-specific artificial selection on the fighting legs of male *Rhizoglyphus echinopus* over 13 generations. The rearing conditions and selection protocol are described in detail by Buzatto *et al*.^12^. In brief, we started each selection line with 75 families composed of virgin adults (one fighter male and three females) that had been reared in isolation. We assigned 25 families to each of three replicate lines (A, B and C). After a mating period of 2–4 days, males were mounted on microscope slides and we measured the width of their third right leg, and the anterior coxae suture (ACS) as a proxy of body size, following Pike *et al*.^10^. Using type I linear models to fit the relationship between these traits, we selected the five males in each line with the largest (up lines) or smallest (down lines) residuals from these models to create six replicate lines (*A*_*up*_, *A*_*down*_, *B*_*up*_, *B*_*down*_, *C*_*up*_ and *C*_*down*_). 40 offspring of each selected male were reared in isolation to produce virgin adults for the following generations. The virgin adults were scored for sex and morph to produce our sex and morph ratio data. Next, out of these adults, 20 fighters (per line) were paired with two unrelated females from the same line. This selection regime was followed for the next 12 generations, but using separate linear models for each line, and selecting five males out of a pool of 20 per generation. Throughout the experiment, individuals were always reared in isolation in cylindrical glass vials (diameter = 10 mm, height = 14 mm) and mated in plastic tubes (diameter = 25 mm, height = 14 mm). Both individual vials and mating tubes had a plaster-of-Paris base 3 mm thick that was kept damp by placing them on a piece of damp filter paper in a Petri dish. All vials and tubes were kept inside round plastic containers (diameter = 76 mm, height = 40 mm), kept in dark incubators at 22 °C. The results of leg morphology evolution in these lines are reported in^12^.

Here we report the results of a new fecundity trial that we started after generation 13. In each replicate line, we isolated 40 offspring from each of the five paternal families composed of half-sibs of a fighter male and two unrelated females (equivalent to the selection protocol described above). We then randomly selected 20–25 virgin females (i.e. 5 daughters per paternal families, according to the availability of females in each family). After a 2-day maturation period, where females were kept in isolation in individual vials (as described above) with *ad libitum* dried yeast for food, we paired them to two unrelated scrambler males from our stock populations. These families were kept in the mating tubes (also described above) with *ad libitum* dried yeast. Only one male morph (scramblers) was used here because we know that paternal effects act on morph ratio in the species^30^, and we wanted to avoid confounding effects of differential paternal effects in our results. After 7 days, during which females mated and started to lay eggs (following^16^), we froze all families to then count the total number of eggs laid. The number of eggs laid in the first seven days is a good estimate of a female’s lifetime fecundity in *Rhizoglyphus* mites^31^, and pairing the female with two males ensures that her fecundity is not limited by sperm depletion. Females that died before the end of the egg laying period were removed from the analyses, but females that lived but failed to lay eggs were included, since these failures could indicate an extreme fecundity cost due to infertility. We analyzed the data by fitting two generalized mixed effects models (with Poisson errors) to the fecundity of females, one with just an intercept, and the other with the fixed effect of selection direction. In both models, selection line ID was a random effect. We then compared these models using the bias-corrected version of the Akaike Information Criterion (AIC_c_).

We tracked morph ratio and sex ratio throughout the experiment, and analysed their evolution by fitting separate generalized mixed effects models for sex and morph ratio in different sets of candidate models with binomial errors. We used data from the first nine generations of selection — after generation 10 we moved our lines to a 10 °C incubator to increase generation time for logistic reasons. Since temperature affects the proportion of fighters^32^, we only report sex and morph ratio evolution until generation nine, while the lines were kept at 22 °C. It is important to note however that the potential effects of temperature on ratios (and on female fecundity as well) would be the same for up and down lines, as they were all submitted to the same temperature changes. For the analyses, we built a set of five candidate models with the combinations of the fixed effects of generation, selection direction and their interaction, fitting all models with maximum likelihood through Laplace approximation. We again compared models on the basis of their AIC_c_. We also added observation-level random effects^33^ to account for the overdispersion detected when comparing the sum of squared Pearson residuals to the models’ residual degrees of freedom. We visually checked all our models for heteroscedasticity of residuals. All analyses were performed in R, version 3.5.0^34^, using packages *lme4*^35^ and *AICcmodavg*^36^.

## Data Availability

Data is available at Dryad (10.5061/dryad.nzs7h44n5).

## References

[1] Lande R (1980). Sexual dimorphism, sexual selection, and adaptation in polygenic characters. Evolution.

[2] Williams TM, Carroll SB (2009). Genetic and molecular insights into the development and evolution of sexual dimorphism. Nat. Rev. Genet..

[3] Oliveira, R. F., Taborsky, M. & Brockmann, H. J. *Alternative Reproductive Tactics: an Integrative Approach*. (Cambridge University Press, 2008).

[4] Tomkins JL, Hazel WN (2007). The status of the conditional evolutionarily stable strategy. Trends Ecol. Evol..

[5] Rice WR (1984). Sex-chromosomes and the evolution of sexual dimorphism. Evolution.

[6] Rice WR, Chippindale AK (2001). Intersexual ontogenetic conflict. J. Evol. Biol..

[7] Simpson SJ, Sword GA, Lo N (2011). Polyphenism in insects. Curr. Biol..

[8] West-Eberhard, M. J. *Developmental Plasticity and Evolution*. 794 (Oxford University Press, 2003).

[9] Emlen DJ, Hunt J, Simmons LW (2005). Evolution of sexual dimorphism and male dimorphism in the expression of beetle horns: Phylogenetic evidence for modularity, evolutionary lability, and constraint. Am. Nat..

[10] Pike KN, Tomkins JL, Buzatto BA (2017). Mixed evidence for the erosion of intertactical genetic correlations through intralocus tactical conflict. J. Evol. Biol..

[11] Morris MR, Goedert D, Abbott JK, Robinson DM, Rios-Cardenas O (2013). Intralocus tactical conflict and the evolution of alternative reproductive tactics. Adv. Study Behav..

[12] Buzatto Bruno A., Clark Huon L., Tomkins Joseph L. (2018). Morph-specific artificial selection reveals a constraint on the evolution of polyphenisms. Proceedings of the Royal Society B: Biological Sciences.

[13] Radwan J, Unrug J, Tomkins JL (2002). Status-dependence and morphological trade-offs in the expression of a sexually selected character in the mite, *Sancassania berlesei*. J. Evol. Biol..

[14] Tomkins JL, Hazel WN, Penrose MA, Radwan J, LeBas NR (2011). Habitat complexity drives experimental evolution of a conditionally expressed secondary sexual trait. Curr. Biol..

[15] Harano T, Okada K, Nakayama S, Miyatake T, Hosken DJ (2010). Intralocus sexual conflict unresolved by sex-limited trait expression. Curr. Biol..

[16] Plesnar-Bielak A, Skrzynecka AM, Miler K, Radwan J (2014). Selection for alternative male reproductive tactics alters intralocus sexual conflict. Evolution.

[17] Tomkins JL, Radwan J, Kotiaho JS, Tregenza T (2004). Genic capture and resolving the lek paradox. Trends Ecol. Evol..

[18] Emlen DJ (2008). The Evolution of Animal Weapons. Annu. Rev. Ecol. Evol. Syst..

[19] Emlen DJ, Warren IA, Johns A, Dworkin I, Lavine LC (2012). A mechanism of extreme growth and reliable signaling in sexually selected ornaments and weapons. Sci..

[20] Honěk A (1993). Intraspecific variation in body size and fecundity in insects: a general relationship. Oikos.

[21] Buzatto BA, Buoro M, Hazel WN, Tomkins JL (2015). Investigating the genetic architecture of conditional strategies using the Environmental Threshold Model. Proc. R. Soc. B-Biol Sci..

[22] Buzatto BA, Simmons LW, Tomkins JL (2012). Genetic variation underlying the expression of a polyphenism. J. Evol. Biol..

[23] Radwan J (2009). Alternative mating tactics in acarid mites. Adv. Study Behav..

[24] Unrug J, Tomkins JL, Radwan J (2004). Alternative phenotypes and sexual selection: can dichotomous handicaps honestly signal quality?. Proc. R. Soc. B-Biol Sci..

[25] Grondziel E (1975). Sex chromosomes in *Rhizoglyphus echinopus* (F, & R.) (Acarina, Acaridae). Folia Biologica.

[26] Trivers, R. L. & Willard, D. E. Natural selection of parental ability to vary sex ratio of offspring. *Science* **179**, 90–92 (1973). 10.1126/science.179.4068.90 4682135

[27] Booksmythe I, Mautz B, Davis J, Nakagawa S, Jennions MD (2017). Facultative adjustment of the offspring sex ratio and male attractiveness: a systematic review and meta-analysis. Biol. Rev. Camb. Philos. Soc..

[28] Macke E (2011). Sex allocation in haplodiploids is mediated by egg size: evidence in the spider mite *Tetranychus urticae* Koch. Proc. R. Soc. B-Biol Sci..

[29] Eberhard WG (2018). Sexual selection and static allometry: the importance of function. Q. Rev. Biol..

[30] Buzatto BA, Simmons LW, Tomkins JL (2012). Paternal effects on the expression of a male polyphenism. Evolution.

[31] Konior M, Radwan J, Kolodziejczyk M (2001). Polyandry increases offspring fecundity in the bulb mite. Evolution.

[32] Radwan J (2001). Male morph determination in *Rhizoglyphus echinopus* (Acaridae). Exp. Appl. Acarology.

[33] Harrison XA (2014). Using observation-level random effects to model overdispersion in count data in ecology and evolution. PeerJ.

[34] R: A language and environment for statistical computing. (R Foundation for Statistical Computing, Vienna, Austria, 2018).

[35] Bates, D., Maechler, M., Bolker, B. & Walker, S. Fitting linear mixed-effects models using *lme4*. *J Stat Softw* **67**, 1–48, http://CRAN.R-project.org/package=lme4 (2015).

[36] Mazerolle, M. J. AIC: Model selection and multimodel inference based on (Q)AIC(c). *R package version 2.0-1*, http://CRAN.R-project.org/package=AICcmodavg (2014).

